# Crossing Methods and Cultivation Conditions for Rapid Production of Segregating Populations in Three Grain Amaranth Species

**DOI:** 10.3389/fpls.2016.00816

**Published:** 2016-06-07

**Authors:** Markus G. Stetter, Leo Zeitler, Adrian Steinhaus, Karoline Kroener, Michelle Biljecki, Karl J. Schmid

**Affiliations:** Institute of Plant Breeding, Seed Science and Population Genetics, University of HohenheimStuttgart, Germany

**Keywords:** amaranth, hybridization, hot water emasculation, hand emasculation, genetic resources, marker assisted breeding

## Abstract

Grain amaranths (*Amaranthus* spp.) have been cultivated for thousands of years in Central and South America. Their grains are of high nutritional value, but the low yield needs to be increased by selection of superior genotypes from genetically diverse breeding populations. Amaranths are adapted to harsh conditions and can be cultivated on marginal lands although little is known about their physiology. The development of controlled growing conditions and efficient crossing methods is important for research on and improvement of this ancient crop. Grain amaranth was domesticated in the Americas and is highly self-fertilizing with a large inflorescence consisting of thousands of very small flowers. We evaluated three different crossing methods (open pollination, hot water emasculation and hand emasculation) for their efficiency in amaranth and validated them with genetic markers. We identified cultivation conditions that allow an easy control of flowering time by day length manipulation and achieved flowering times of 4 weeks and generation times of 2 months. All three different crossing methods successfully produced hybrid F_1_ offspring, but with different success rates. Open pollination had the lowest (10%) and hand emasculation the highest success rate (74%). Hot water emasculation showed an intermediate success rate (26%) with a maximum of 94% success. It is simple to perform and suitable for a more large-scale production of hybrids. We further evaluated 11 single nucleotide polymorphism (SNP) markers and found that they were sufficient to validate all crosses of the genotypes used in this study for intra- and interspecific hybridizations. Despite its very small flowers, crosses in amaranth can be carried out efficiently and evaluated with inexpensive SNP markers. Suitable growth conditions strongly reduce the generation time and allow the control of plant height, flowering time, and seed production. In combination, this enables the rapid production of segregating populations which makes amaranth an attractive model for basic plant research but also facilitates further the improvement of this ancient crop by plant breeding.

## 1. Introduction

Ancient crops from the Americas such as quinoa (*Chenopodium quinoa* willd.) or amaranth (*Amaranthus* spp. L.) are a valuable addition to the human diet because of their high nutritional value. These pseudocereals have a high protein content and are rich in lysine and other essential amino acids that are limited in other grains (Vega-Gálvez et al., 2010; Rastogi and Shukla, 2013). In addition, these crops are well adapted to harsh environmental conditions and are therefore suitable for cultivation on marginal soils. Their yields are significantly lower than those of major crops due to a lack of plant breeding (Reta Alemayehu et al., 2015), but the presence of a high genetic and phenotypic diversity in these species indicates an excellent potential for breeding and variety development (Brenner et al., 2010).

Grain amaranth originated from Central and South America, where it was of great importance in pre-columbian agriculture until its cultivation strongly declined after the Spanish conquest (Sauer, 1967; Kauffman and Weber, 1990; Brenner et al., 2010). Three species of *Amaranthus* are cultivated for grain production: *A. caudatus* L., *A. cruentus* L., and *A. hypochondriacus* L. Amaranth expresses the C_4_ carbon cycle, which is more common in grasses but rare in dicots. Despite a high genetic diversity (Stetter et al., 2015), breeding efforts in amaranth so far were limited to the selection of suitable genotypes from landraces. Amaranth is mainly self-pollinating and has numerous intricate flowers, which make crosses more difficult than in other crops. The ability to efficiently carry out crosses is an important requirement for plant research to understand genetic basis of relevant traits (Moose and Mumm, 2008; Olsen and Wendel, 2013). Crosses are equally important for plant breeding and are used to generate new genetic variation and to introgress exotic material into breeding populations.

In many crops, hybrid varieties are characterized by strongly increased yields (Duvick, 2001). The application of hybrid breeding in amaranth is also very promising, because a mid-parent heterosis of up to 88% has been reported (Lehmann et al., 1991). The ability to conduct crosses on a large scale with little effort is of central importance for the development and production of hybrid crop varieties. To use this potential in minor crops, an improvement of crossing methods is essential (Veerappan et al., 2014).

**Figure 1:**
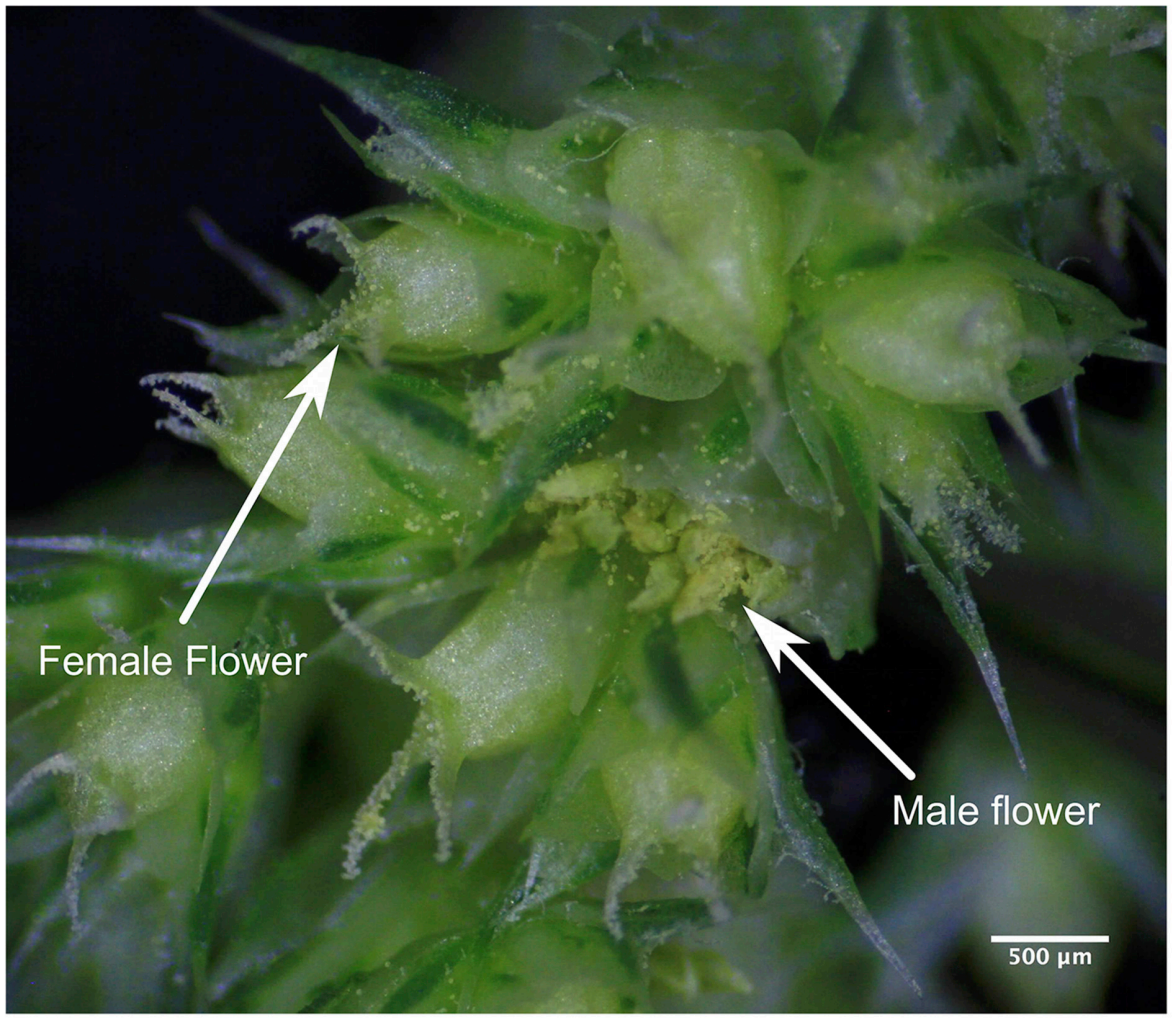
**Flower morphology**. Inflorescence of *A. caudatus* consisting of flower clusters in which a male flower in the center is surrounded by several female flowers.

Several approaches for hybrid production are available, but for all methods the key step is to prevent self-fertilization by the male parent. This is either by using appropriate genetic self-incompatibility systems or by mechanical and chemical treatments that lead to male sterility. In several species, cytoplasmatic male sterility (CMS) systems prevent selfing of the female crossing partner (Laser and Lersten, 1972). To use CMS systems for breeding male sterile female parent and male parents with restorer genes are needed to allow seed production in the hybrid progeny. Additionally, a maintainer line is needed that allows multiplying the male sterile line without loosing the CMS. Male sterility has been reported in *A. hypochondriacus* but is not yet developed sufficiently to be used for breeding (Peters and Jain, 1987). Mechanical emasculation methods are efficient if the male and female flower are well separated on the plant (e.g., as in maize) because then male flowers can be removed without interfering with the female inflorescence. In other crops like tomato and *Medicago*, anthers are removed before pollen shedding (Veerappan et al., 2014). Another physical method is the heat treatment of the flowers of the female parent to destroy the pollen, for example by a hot water treatment. Here, the temperature is crucial, as differences by few degrees can influence the efficiency of the emasculation (Mukasa et al., 2007; García-Yzaguirre and Carreres, 2008; Otsuka et al., 2010). Chemical gametocides are used in hermaphrodite crops for which no CMS systems are available or are too costly, for example in wheat (Dotlacil and Apltauerová, 1978). The grain amaranth species have male and female flowers on the same inflorescence where several female flowers are arranged circularly around a male flower (Figure 1). The flowers are less than 1 mm in diameter, which makes mechanical emasculation difficult. For this reason other emasculation methods such as a hot water treatment may be more efficient.

Frequently, crossing methods are not completely reliable and require the validation of progeny. Phenotypic traits with a dominant-recessive inheritance can be used to identify successful crosses. In amaranth, traits such as seed or leaf color differ between genotypes and are available for validation (Kulakow et al., 1985). For phenotypic traits to be useful, however, parents need to differ in at least one trait and the male parent needs to express the dominant allele. In contrast, molecular markers allow an efficient and early evaluation of crosses without restricting the combination of parents, and cost-efficient PCR-based marker systems are available for this purpose (Maughan et al., 2011).

For model plants it is important to take specific requirements of development into account. Amaranth shows a strong photoperiod sensitivity and starts to flower under short day conditions (Brenner et al., 2010). A single plant has the potential to produce several thousands of seeds and can therefore produce large populations. However, under field conditions amaranth plants are usually tall and require a significant amount of space for cultivation. If flowering time, plant size and seed production can be controlled in climate chambers and greenhouses, an efficient propagation of the plant may be possible.

In the work presented here we study the efficiency of three different crossing methods and describe environmental conditions in a controlled environment (growth chamber) to achieve efficient and rapid generation of progeny for genetic studies. We suggest a method for hybrid identification with cost efficient PCR- based markers. Subsequently, we apply our method to three species of amaranth to evaluate its potential for the wider application to species within the genus *Amaranthus*.

## 2. Methods

### 2.1. Plant material and growth conditions

The amaranth accessions for testing the three crossing methods were selected to comprise accessions with green seedlings as female parent and accessions with red seedlings as male parent. Additionally, amaranth varieties were used to verify hybridization and the use of genetic markers (Table 1 and Table S2). Single seeds were planted in 7 × 7 cm pots in standard gardening soil. Plants were grown for 2 weeks under long day conditions (Table 2) before transferring them step-wise in weekly intervals to short day conditions (Table 2). This helped to synchronize flowering of different genotypes and spread workload for performing the crosses.

**Table 1:** **Parental genotypes for 11 KASP marker assays**.

**ID**	**Name**	**Species**	**AM17978**	**AM19584**	**AM19963**	**AM21336**	**AM21605**	**AM22029**	**AM22341**	**AM24451**	**AM24579**	**AM25548**	**AM26171**
26	PI 642741	*A. caudatus*	FAM	HEX	HEX	HEX	HEX	HEX	FAM	FAM	-	HEX	HEX
34	PI 511679	*A. caudatus*	FAM	HEX	FAM	HEX	FAM	HEX	HEX	HEX	FAM	HEX	HEX
37	PI 649220	*A. caudatus*	FAM	HEX	FAM	HEX	FAM	HEX	FAM	FAM	FAM	HEX	HEX
117	PI 511684	*A. hybr.*	FAM	HEX	FAM	HEX	FAM	HEX	HEX	FAM	FAM	HEX	HEX
174	PI 649623	*A. hypochondriacus*	HEX	FAM	FAM	-	FAM	FAM	HEX	HEX	HEX	FAM	FAM
245	Baernkrafft	*A. cruentus*	FAM	FAM	FAM	FAM	FAM	HEX	HEX	HEX	HEX	FAM	HEX
246	C6	*A. cruentus*	FAM	HEX	FAM	FAM	FAM	HEX	HEX	HEX	HEX	FAM	HEX
247	Puerto Moutt	*A. cruentus*	FAM	FAM	FAM	FAM	FAM	HEX	HEX	HEX	HEX	FAM	HEX
248	Pastewny	*A. hybridus*	-	FAM	FAM	-	FAM	FAM	HEX	HEX	HEX	-	FAM
369	PI 511695	*A. caudatus*	FAM	HEX	HEX	HEX	HEX	HEX	FAM	FAM	FAM	HEX	HEX

HEX and FAM are the fluorescence dyes associated with each allele. Markers were tested in 10 individuals of which 8 were used for crosses.

**Table 2:** **Growth conditions**.

	**Day length**	**Light intensity**	**Temp day**	**Temp night**
Long day	16	150 mmol	35°C	30°C
Short day	8	150 mmol	30°C	25°C

Parameters for amaranth in growth chamber for long and short day conditions.

### 2.2. Crossing methods

**Figure 2:**
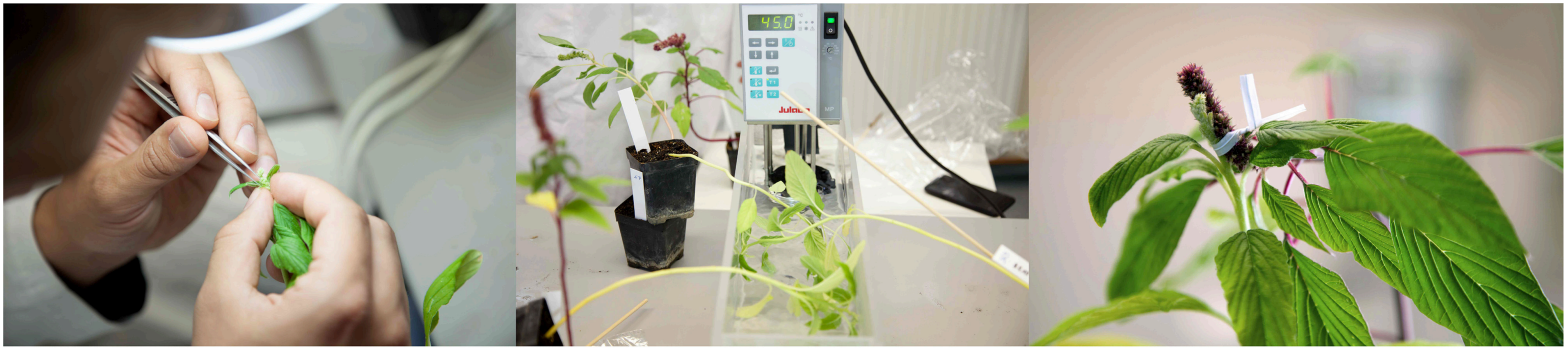
**Crossing methods**. Three crossing methods: **(A)** Hand emasculation by removal of male flowers from female plant. **(B)** Hot water emasculation by 10 min treatment with 45°C water bath. **(C)** Fixing male and female flower to each other for better pollen transfer.

We evaluated three different methods for crossing wild (*A. hybr.* and *A. hybridus*) and cultivated species of amaranth (Figure 2 and Table 1). The first method was open pollination by fixing the flowers of the female and male parent to each other and protecting them with a pollen proof bag (Sealed Air, Germany) from cross pollination by other plants. The second method was a warm water treatment of the inflorescence during flower initiation of the first emerging flowers (García-Yzaguirre and Carreres, 2008). Female flowers were dipped into a water bath of 45°C warm water for 10 min to emasculate the male flowers before proceeding as in the first method. The water treatment was repeated after 7 days. The third method was hand emasculation. For this approach, female flowers that were already open and all male flowers were removed from the inflorescence. The tip of the inflorescence was also removed to prevent the emergence of new flowers. The emasculation was repeated after 7 days and any flowers that developed later were removed. For all three methods plants were shaken daily to increase pollen dispersal and to assure cross-fertilization.

### 2.3. Success evaluation and statistical analysis

Seeds of the female parent were harvested 4 weeks after crossing. For each cross 50 seeds were counted and planted in pots. Seedling color evaluation was performed 2 weeks after planting by counting green and red seedlings. The success rate was the ratio between red and green offspring. Data analysis to test the differences between methods and between crossing types was conducted with a Generalized Linear Model (GLM) with binomial variance and a logit link function that included the crossing method (α), the crossing type (β, Intra- and inter-specific) and the interaction as factors:

(1) $$\begin{array}{l} logit\left(\mu_{ij}\right)=log\left(\frac{\mu_{ij}}{1-\mu_{ij}}\right)=\eta_{ij}=\mu +\alpha_{i}+\beta_{j}+{\left(\alpha \beta \right)}_{ij}. \end{array}$$

The calculation was done with the R statistical package version 3.2.0 using the stats library.

### 2.4. DNA extraction

For genotyping the DNA was extracted with EconoSpin columns (Epoch Life Science Inc.) using 1% CTAB extraction buffer (Saghai-Maroof et al., 1984). Dry leaf samples were homogenized and incubated for 2 h at 50°C in 400 μl 1% CTAB extraction buffer and 4 μl Proteinase K. After addition of 300 μl Ammonium acetate (7.5 M) and 300 μl Ethanol (96%), the samples were centrifuged for 1 min at full speed. Then 800 μl of the supernatant were transferred on a EconoSpinÂő column placed in the collection tube and centrifuged for 1 min. The flow through was discarded. The columns were washed twice with wash buffers from Saghai-Maroof et al. (1984) before eluting DNA twice with 50 μl Tris-HCl (10 mM, pH 8).

### 2.5. Evaluation of genetic markers

Eleven KASP assays (LGC Berlin/Germany) were selected from Maughan et al. (2011) to validate crosses (Table S1). The assays were prepared with 5 μl DNA (10 ng/μl) and 5 μl genotyping mix and run on the LightCycler 480 Instrument II (Roche Life Science) with standard settings as given by the KASP manual (LGC Berlin/Germany) and analyzed using the LightCycler 480 Software. First, parental lines were evaluated to find polymorphic markers for each of the crosses. Later, these markers were used to validate the crosses. For a proof of concept we genotyped offspring that were evaluated before by their seedling color. Both offspring with green (selfed plants) and red (hybrids) were genotyped.

### 2.6. Additional hybrid production

The previously evaluated hand emasculation method was used to produce additional hybrids. Plants were grown as described above, but crossing partners were not restricted to different seedling colors. The success of the crosses was validated with SNP markers.

## 3. Results and discussion

### 3.1. Cultivation and life cycle

In the field the generation time of the three grain amaranth species is approximately 6 months and leads to very tall plants with thousands of flowers. To reduce the generation time, plant height and number of flowers, we cultivated the plants under short day conditions (8 h) and high temperature (30°C) which both induced early flowering 4 weeks after planting. Additionally, we controlled the initiation of flowering by transferring plants from long day (16 h, 35°C) conditions to short day conditions. Under long day conditions the plants displayed strong vegetative growth and did not flower within 10 weeks after planting, but started flowering approximately 14 days after a transfer to short day conditions. The step-wise transfer of plants from long to short-day conditions allows the production of plants in different flowering stages, which greatly facilitates synchronous flowering for crosses between genotypes that differ in their flowering time. This treatment is further useful to produce male parents that are able to shed large amounts of pollen when females parents start flowering. As soon as 4 weeks after flowering, mature seeds could be harvested. By employing these treatments, very short generation times can be achieved that allow up to six generations per year, which is comparable to the model plant *Arabidopsis thaliana*. In addition, plant height and seed number per plant can be controlled by adjusting growth conditions. Long day conditions lead to more vegetative growth, later flowering and more seeds, while short day conditions result in small early flowers. This is useful for different applications, because crosses require only few flowers, whereas the resulting F_1_ plants should produce larger amounts of seeds (e.g., for creating mapping populations).

### 3.2. Crossing methods

We compared three crossing methods that included open pollination, hot water emasculation and hand emasculation. All three methods produced successful crosses, but the success rates and variances differed strongly between the methods (Table 3). The amount of seeds produced did not substantially differ between methods and the mother plants produced between 100 and 200 seeds. Open pollination between two plants under a single bag without emasculation of the female parent led to a mean success rate of 10% with a standard deviation (s.d.) of 0.05. The hot water treatment of the female parent led to a significantly increased success rate of 26% but with a very high deviation (s.d. = 0.35) and a minimal success rate of 0%. However, the maximal success after hot water treatment was 94%, which shows that the method has a high potential if the key conditions for a successful application can be identified. We sterilized flowers at 45°C and an adaptation of temperature may contribute to a higher rate of success. In other species (e.g., Acacia, buckwheat and rice), different temperatures change the efficiency of emasculation (Mukasa et al., 2007; García-Yzaguirre and Carreres, 2008; Otsuka et al., 2010). A temperature of 45°C for emasculation is rather high compared to other crops (García-Yzaguirre and Carreres, 2008), but not too high because the amaranth plants still set seeds after this treatment and a further optimization may be achieved by varying the length of the heat treatment. Overall, hot water emasculation works with amaranth and, if it can be further improved, is suitable for application in the field to large numbers of plants.

**Table 3:** **Success rate of different crossing methods**.

	**Type**	***N***	**Mean (%)**	**SD (%)**	**Minimum (%)**	**Maximum (%)**
Open pollination		7	10^c^	5	4	18
	Intra-specific	3	11	3	8	14
	Inter-specific	4	10	6	4	18
Heat treatment		8	26^b^	35	0	94
	Intra-specific	4	26	45	0	94
	Inter-specific	4	27	27	0	57
Hand emasculation		11	74^a^	29	17	100
	Intra-specific	4	80	20	50	94
	Inter-specific	7	71	34	17	100

Success rates and standard deviation (SD) for different crossing methods based on seedling color of 50 offspring per sample. The mean was calculated on basis of four to seven crosses (N) per method and crossing type. Intra-specific crosses were performed with A. caudatus (PI 511679 × PI 649220) and inter-specific crosses between A. caudatus (PI 511679) and A. hybr. (PI 511684). A Generalized Linear Model (GLM) with binomial variance and a logit link function were used to analyze differences between methods. Different letters show significant differences between methods. There was no significant difference between intra- and inter- specific crosses.

**Figure 3:**
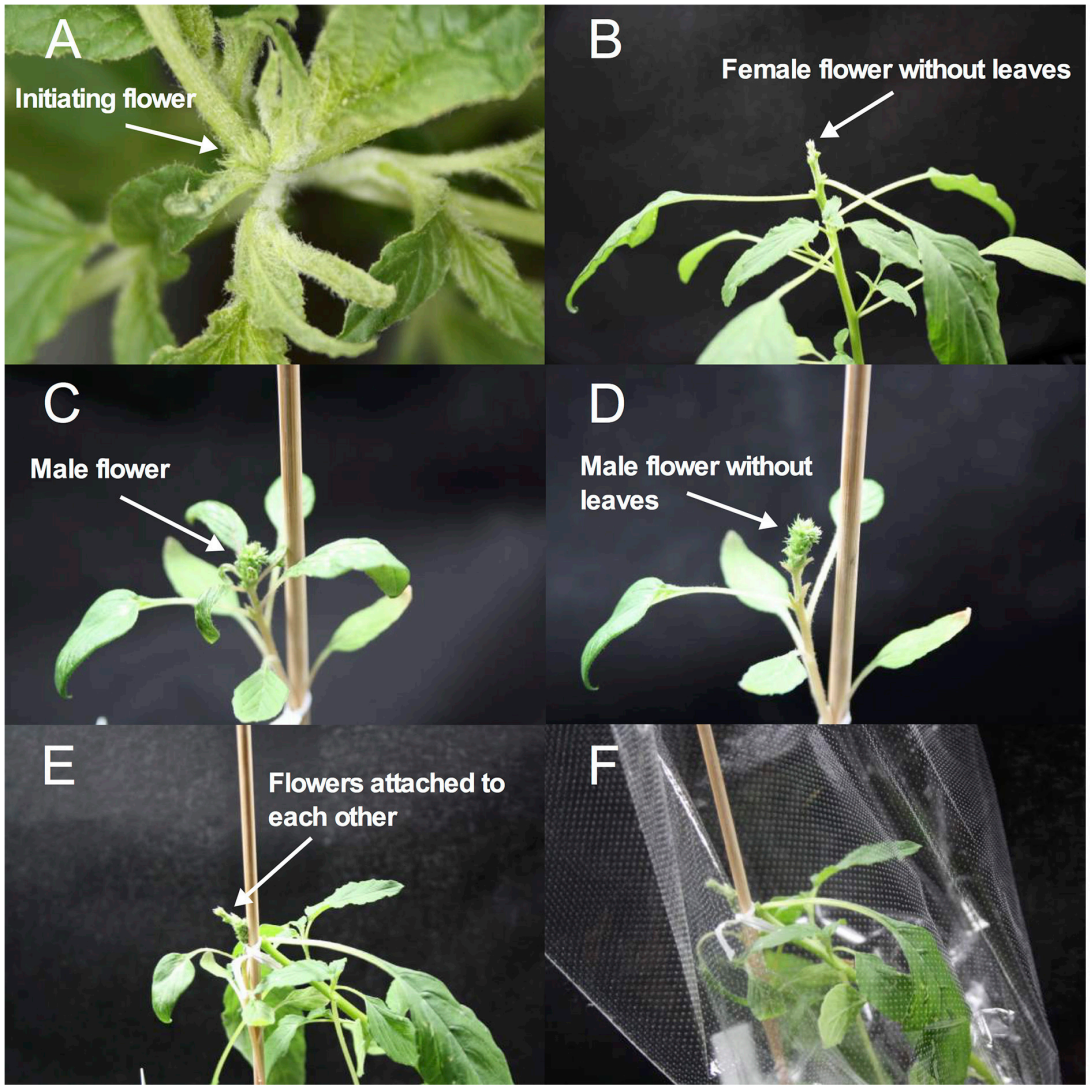
**Hand crossing procedure. (A)** Flower initiation of female plant. **(B)** Female plant prepared for crossing. Leaves near the flower are removed. **(C)** Male crossing partner with first open male flowers. **(D)** Male plant prepared for crossing. Leaves near the flower are removed for improved pollen exchange. **(E)** Female and male crossing partners attached to each other. **(F)** Crossing partners are isolated with pollen proof bag to avoid contamination by foreign pollen.

The most elaborated and time consuming method we evaluated was hand emasculation (Figure 3). The mean success rate of 74% was the highest of the three methods and the deviation (s.d. = 0.29) was lower than of the heat treatment. The minimum success was comparable to free pollination, but the maximum success was up to 100%. Hand emasculation is difficult because amaranth has many small flowers and each male flower sheds enough pollen to pollinate a whole plant. Therefore it is critical to remove all male flowers from the female parent before flower dehiscence. The deviation can be decreased by keeping only few flower clusters per plant. We also tested whether intra- and inter-specific crosses are different in their efficiency, but there was no significant difference between intra- and inter-specific crosses (Table 3). This shows that inter-specific hybridization is possible, but as the two species are closely related this might not be the case for distant member of the *Amaranthus* genus.

The comparison of the three methods shows that open pollination had low success rates, whereas heat treatment can be an effective and simple method for crosses if many seeds are required and simple morphological markers are available for the evaluation of offspring. Hand emasculation by well trained personnel shows the best performance. Since amaranth plants may produce thousands of seeds, a single successful cross can produce large F_2_ populations, and the number of hand crosses needed can be kept low, which decreases the work load of the method substantially and makes it suitable for large projects.

### 3.3. Genetic markers for hybrid identification

Since no crossing method provides a 100% success rates, unsuccessful crosses have to be excluded in early stages. Furthermore, crossing partners should not be limited by phenotypic differences in certain traits (e.g., different seedling color), but all possible combinations parents should be available. We therefore evaluated all accessions used in this study with 11 PCR-based SNP markers. The markers were the most polymorphic from a set of 411 KASP markers from Maughan et al. (2011). Each marker was polymorphic between at least two lines and each cross segregated at least for one marker (Table 1). After evaluating the parental lines, we selected suitable markers to evaluate crosses.

**Figure 4:**
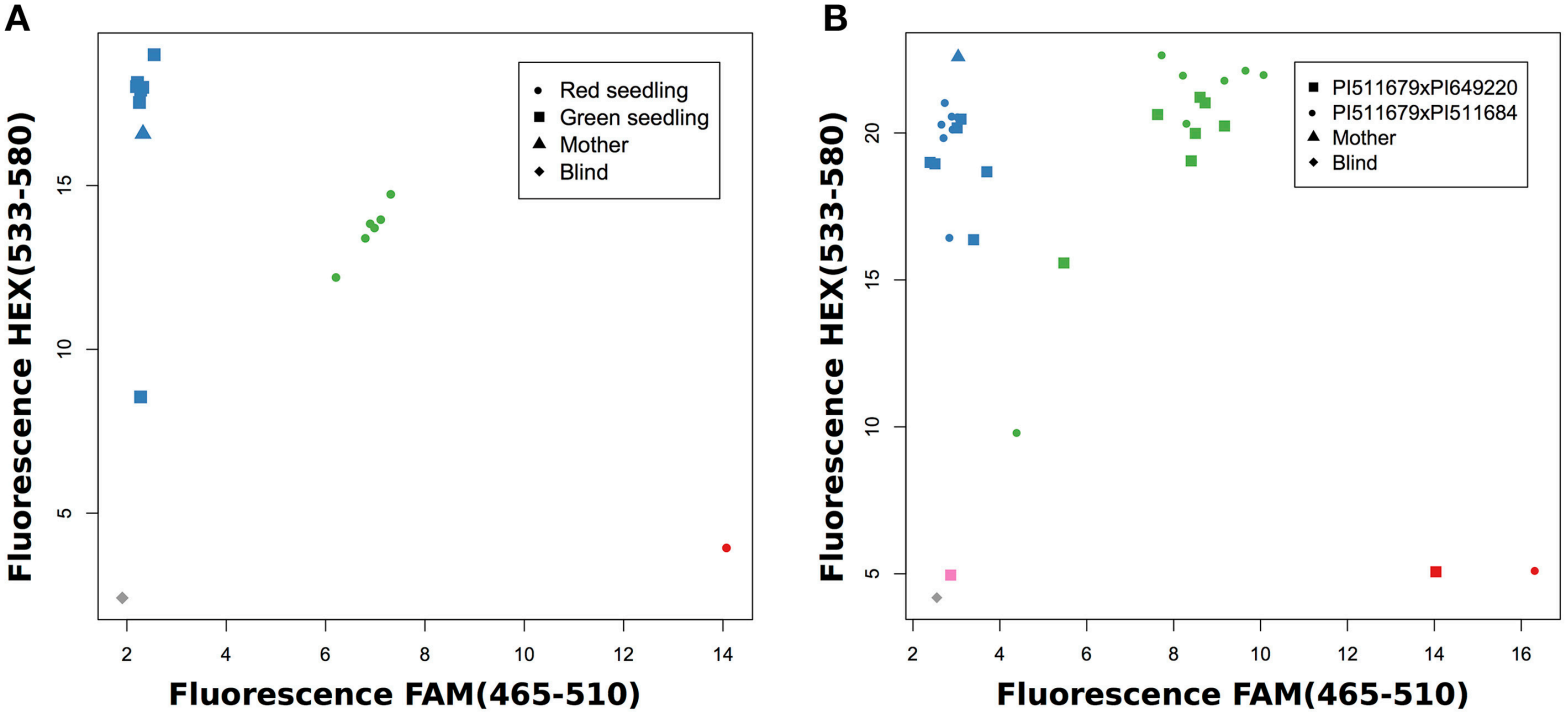
**SNP genotyping for known crosses. (A)** Validation of PI511679xPI649220 with AM22341 and comparison with seedling color. **(B)** Validation of two crosses with AM24451.

First, we investigated progeny which had already been evaluated by their seedling color, because we expected green seedlings to be homozygous for the maternal allele since the green allele is recessive, and red seedlings to be heterozygous. For example, the application of marker AM22341 in a cross of PI511679 × PI649220 showed that green seedlings were homozygous for the allele of parent PI511679 and red seedlings were heterozygous for both parental alleles (Figure 4A). Frequently, the same marker can be used in several crosses, which allows the evaluation of more than one cross simultaneously (Figure 4B). This strongly decreases the work load and the cost of the evaluation. When working with homozygous parental lines a single maker is sufficient to validate successful crosses.

To test the effectiveness of the hand crossing method and the validation with genetic markers, we produced hybrids between amaranth genotypes from different species and validated them with the marker system. Although not all crosses produced hybrids, for most crosses the number of hybrids produced was high and less than 10 offspring had to be evaluated per cross (Table 4).

**Table 4:** **Crosses of different amaranth varieties by hand emasculation and evaluation of success rates with SNP markers**.

	**ID (Mother)**	**ID (Father)**	**Marker**	**Genotyped**	**Selfings**	**Successful crosses**	**Failed assay**
1	34	245	AM19584	7	0	7	0
2	34	245	AM19584	6	2	2	2
3	34	245	AM19584	7	2	4	1
4	34	248	AM19584	7	2	4	1
5	34	248	AM19584	7	0	6	1
6	34	248	AM19584	7	0	4	3
7	245	26	AM19584	6	4	2	0
8	245	26	AM19584	8	2	3	3
9	247	248	AM22029	10	1	9	0
10	247	248	AM22029	9	9	0	0
11	248	245	AM22029	6	6	0	0
12	248	245	AM22029	8	5	0	3
13	248	245	AM22029	7	4	0	3

All crosses are interspecific crosses between the three grain amaranths and/or their putative ancestors. The ID of mothers and fathers corresponds to Table 1.

## 4. Conclusions

Ancient and underutilized crops greatly benefit from the ongoing revolution in genomics. However, to utilize this information for the improvement of minor crops, efficient crossing methods which are the basis of breeding programs need to be established. We developed crossing methods and genetic markers for hybrid identification in amaranth and showed that these can be used for crosses within and between species. We further showed that the life cycle and plant size of amaranth can be reduced substantially when light and temperature conditions are adapted. For genetic and physiological studies a short generation time is advantageous, which is a common characteristic of model organisms for basic research. Under the conditions described here, generation times as short as those of *A. thaliana* are possible (Meyerowitz and Pruitt, 1985). Additionally, the amount of seeds can be controlled, which allows the production of large offspring populations for genetic mapping. Furthermore, amaranth has a relatively small genome (500 Mbp) with a reference sequence, and a large number of genotyped genebank accessions are available (Stetter et al., 2015; Clouse et al., 2016). Taken together, these resources and the possibility of interspecific crosses make the grain amaranth species a very suitable model organism for studying fundamental processes such as adaptation, speciation, heterosis, C_4_ photosynthetic metabolism, or domestication. The ability to conduct crosses from genetically diverse material facilitates the establishment of advanced breeding programs and the selection of improved genotypes using current breeding methods such as genomic selection will improve the value of this minor crop for agricultural production.

## Author contributions

KS and MS designed the experiments. LZ, AS, KK, and MB performed the crosses. MS performed the genotyping and analyzed the data. KS and MS wrote the manuscript. All authors read and approved the final manuscript.

### Conflict of interest statement

The authors declare that the research was conducted in the absence of any commercial or financial relationships that could be construed as a potential conflict of interest.
